# Supplementary material for: The genome sequence of the Common Purple & Gold, Pyrausta purpuralis Linnaeus, 1758 (Lepidoptera: Crambidae)
Source: Wellcome Open Res. 2026 Mar 19;10:512. Originally published 2025 Sep 15. [Version 2] doi: 10.12688/wellcomeopenres.24884.2 (PMC13014091; doi:10.12688/wellcomeopenres.24884.2)
Supplement: Peer review history

# Reviewer report: Reviewer response for version 2

**DOI:** 10.21956/wellcomeopenres.28813.r151106

**Published:** 2026-03-24

**Authors:** Annabel Whibley

**Affiliations:** 1 Bragato Research Institute, Lincoln, New Zealand

No further comments. Thank you to the authors for engaging with my earlier review.

Are sufficient details of methods and materials provided to allow replication by others?

Yes

Is the rationale for creating the dataset(s) clearly described?

Yes

Are the datasets clearly presented in a useable and accessible format?

Yes

Are the protocols appropriate and is the work technically sound?

Yes

Reviewer Expertise:

Bioinformatics, Genomics

I confirm that I have read this submission and believe that I have an appropriate level of expertise to confirm that it is of an acceptable scientific standard.

# Reviewer report: Reviewer response for version 1

**DOI:** 10.21956/wellcomeopenres.27409.r135458

**Published:** 2026-02-28

**Authors:** Annabel Whibley

**Affiliations:** 1 Bragato Research Institute, Lincoln, New Zealand

Sims and Colleagues report the genome assembly of the crambid moth *Pyrausta purpuralis* (also known as Common Purple and Gold). The approach follows the Darwin Tree of Life pipelines and reporting templates.  The identification of the specimen was verified by DNA barcoding, a particularly useful check given the challenge in distinguishing congeneric species, as noted by the authors in the background information.  A primary assembly plus supporting alternate contigs are reported. The methods, metadata and genomics resources are of high quality, and extensive evaluation of the resulting assembly is reported.  Public accession links are working. The blob plot includes a large cloud of small contigs, with ah range of GC and coverag values, and which might have been better resolved with deeper  sequencing (although I will note that this is quite a large, highly heterozygous and repeat-rich lepidopteran genome, which does add challenges to assembly). Nevertheless 94% of the assembly sequence was assigned to chromosomal scaffolds.

 The one issue that I have noted is the assertion in the abstract that the sample is a female and which I am struggling to reconcile with the following statement in the assembly statistics: “ The specimen his a homogametic male, with the Z chromosome identified based on homology to the genome of *Pyrausta aurata* (GCA_963584085.1).”  Aside from the typo (“his” should presumably be “is”, I would have expected recovery of a W chromosome in this lineage were the sample female. This is the sole reason I have not approved the report.

Are sufficient details of methods and materials provided to allow replication by others?

Yes

Is the rationale for creating the dataset(s) clearly described?

Yes

Are the datasets clearly presented in a useable and accessible format?

Yes

Are the protocols appropriate and is the work technically sound?

Yes

Reviewer Expertise:

Bioinformatics, Genomics

I confirm that I have read this submission and believe that I have an appropriate level of expertise to confirm that it is of an acceptable scientific standard, however I have significant reservations, as outlined above.

# Reviewer report: Reviewer response for version 1

**DOI:** 10.21956/wellcomeopenres.27409.r135452

**Published:** 2025-10-24

**Authors:** Savarimuthu Ignacimuthu, Sivasankaran Kuppusamy

**Affiliations:** 1 St Xavier's College, Palayamkottai, Tamil Nadu, India; 2 Entomology Research Institute, Loyola College Chennai, Tamil Nadu, India

Authors have done the chromosome-level genome assembly of Common Purple & Gold, *Pyrausta purpuralis* (Linnaeus, 1758). They produced 150.89 Gb of genome through PacBio and Illumina sequencing platforms. Through the assembly 837.24 megabases of genome sequences were received. Authors have used appropriate high molecular weight DNA isolation methods.  Appropriate software was used for the genome annotation and assemblies.

**Comments on the manuscript**

 In the title species describer name and year (Linnaeus, 1758) is not given within parentheses. The name may be given within the parentheses.

 In the Background of the manuscript’s third line, authors have given three species in the genus *Pyrausta*. For the second species, authors have given the full form of the genus name ( *Pyrausta*). The genus name can be in the short form *P. aurata.*

 Authors have used the full form of the genus name *Pyrausta *throughout the text. The genus name can be used in full form first time and can be a short form like *P. purpuralis*

**

 Authors have produced 150.89 Gb of robust genome data. The number of protein-coding genes, number of non-coding genes and number of gene transcripts haven’t been detected through the genome annotation and not listed in the table. Any reason?

 Authors have given the number of scaffolds in the genome assembly statistics table. But the longest scaffold length is given in table 2. 

 Above all, I confirm that the manuscript meets the necessary scientific standard and is suitable for indexing.

Are sufficient details of methods and materials provided to allow replication by others?

Yes

Is the rationale for creating the dataset(s) clearly described?

Yes

Are the datasets clearly presented in a useable and accessible format?

Yes

Are the protocols appropriate and is the work technically sound?

Yes

Reviewer Expertise:

Molecular biology

We confirm that we have read this submission and believe that we have an appropriate level of expertise to confirm that it is of an acceptable scientific standard.
